# Early Management of Paediatric Wrist and Forearm Fractures in a Busy District General Hospital Emergency Department: A Retrospective Cohort Comparison Study and Audit of BOAST Guidelines

**DOI:** 10.7759/cureus.41325

**Published:** 2023-07-03

**Authors:** Benjamin E Fink, Muni T Pinjala, Kudamaduwage P Gomes, William T Mason

**Affiliations:** 1 Trauma and Orthopaedics, Gloucestershire Royal Hospital NHS Foundation Trust, Gloucester, GBR

**Keywords:** orif distal radius, manipulation under anaesthesia, boast, pediatric forearm fracture, distal radius fractures, wrist fractures

## Abstract

Introduction: In response to the strain that the COVID-19 pandemic put on hospitals in the UK, the British Orthopaedic Association, in May 2021, set out British Orthopaedic Association Standards for Trauma and Orthopaedics (BOAST) guidelines for the early management of distal forearm fractures in children. Following this, a local pathway was introduced at our Trust to manage these injuries in the Emergency Department (ED). The aim of this audit was to monitor compliance with the BOAST guidelines and compare the practice with a similar pre-COVID cohort.

Methods: A fixed-date retrospective cohort study was conducted that included cases that presented to the emergency department during a six-month period (August 1, 2021 to January 31, 2022). Data was analysed for rates of primary ED manipulation, documentation of consent and neurovascular status in the notes, orthogonal X-ray data, time till the clinic follow-up, theatre time saved and complications. The ED fracture manipulation rate was also compared with another similar pre-COVID cohort (August 1, 2019 to January 31, 2020) to look for any improvement in the practice.

Results: A total of 86.31% cases were found to have primary fracture manipulation in the ED following the introduction of Trust guidelines in accordance with the BOAST recommendations. This is an improvement in comparison to the 31.94% fracture manipulation rate before the COVID pandemic.

Conclusion: Implementation of the Trust pathway in accordance with the BOAST guidelines along with staff education has standardized the practice at our Trust. It saved approximately 63 hours of trauma theatre time for the six-month data collection period. Our findings also suggest that this has favourable outcomes for the patients with no complications.

## Introduction

Distal radius and forearm fractures (DRFFs) are the most frequent fractures in children, representing up to 36% of total paediatric fractures [1-4]. These fractures are typically caused by fall on an outstretched hand (FOOSH) incidents resulting in angular loading and/or rotational displacement. They are typically classified as physeal injuries of torus (buckle), metaphyseal and forearm shaft types based on the location. Forearm shaft and metaphyseal fractures are either complete or incomplete (greenstick) types [5]. Most of these injuries can be safely managed with non-operative treatment by early manipulation and cast application, which remains the gold standard of care for most fractures. This is possible due to the enormous remodelling potential of the growing skeleton in children. However, some fractures would require complex reduction manoeuvre needing sedation with or without operative interventions (K-wiring, titanium elastic nails, internal fixation with a plate).

In the United Kingdom, the British Orthopaedic Association Standards for Trauma and Orthopaedics (BOAST) has set out guidelines for the early management of DRFFs in children. The new guidelines, published in May 2021, recommend all the NHS Trusts treating these injuries to provide early manipulation and casting as definitive management without the need for admission. The guidelines also suggest the Trusts to have local protocols in place defining the processes of safe procedural analgesia/sedation, allocated safe environment for fracture manipulation, monitoring for complications and recovery from analgesic medication [6,7]. They also emphasize on certain other standards of care in regard to clinical assessment, documentation and follow-up.

## Materials and methods

The audit was conducted at a busy district general hospital (DGH) with paediatric orthopaedic services in the United Kingdom (registered with identification number SG210306). This audit involved a retrospective review of anonymised available data with no patient contact. The UK Policy Framework for Health and Social Care Research tool was used to confirm that it was not a research study and no ethical committee approval was required [8].

Following the publication of the BOAST standards, the Trauma and Orthopaedics and Emergency Department (ED) developed and implemented a local Trust guideline from July 2021 onwards. This Trust guideline was in accordance with the BOAST standards. It has details of a standardized pathway in regard to the following: inclusion and exclusion criteria, recommended analgesia and sedation to be used, specified ED location for safe fracture manipulation and recovery, details on who is required to provide analgesia/sedation and fracture manipulation, guidance regarding types of fractures and manipulation techniques and casting, guidance on acceptable criteria for fracture reduction and referral and follow-up guidance from ED.

Primary outcome measures included the following in the 2021-22 cohort: whether early fracture manipulation was attempted in ED, neuro-vascular status of the limb documentation before and after manipulation, consent documentation in the notes, pre- and post-manipulation orthogonal X-rays, duration for the second review of the patient in a fracture clinic (in days) and failed manipulations and secondary re-manipulation/operative interventions in the theatre. Secondary outcomes included comparison of ED fracture manipulation rates in comparison with a pre-COVID (August 1, 2019 to January 31, 2020) cohort to look for any improvement in practice. This was not a part of the original audit; however, this has been studied to quantify any improvement.

The audit identified children of age 4-16 years who presented to the ED in a six-month period between August 1, 2021 and January 31, 2022, requiring closed reductions of angulated/displaced forearm and wrist fractures. Exclusion criteria encompassed patients with open fractures, neuro-vascular injuries, torus (buckle) fractures, undisplaced fractures, complex fractures that usually require surgery (Monteggia, Galeazzi fractures, complete shaft fractures, fractures with ipsilateral humerus fractures), fractures older than one week and fractures recruited for the CRAFFT randomized study [9].

Data pertaining to a cohort of children from a similar time period, i.e., August 1, 2019 to January 31, 2020 (pre-COVID) was also collected with the same inclusion and exclusion criteria. Patients' hospital medical record numbers (MRNs) collected from the data set were used to find the initial diagnostic plain radiographs in order to radiologically classify the injuries. The MRNs were then used to find the electronic patient notes.

Fisher’s exact test was used for analysis of categorical data; statistical significance was set at p ≤ 0.05 [10].

## Results

In the 2021-22 cohort, 87 children were identified. Overall, the mean age at the time of injury was 9.8 years; the cohort had 51 males and 36 females. Out of the 87 fractures, 73 patients had fracture manipulation in the ED and 14 patients were admitted to the theatre without an attempt of manipulation. Among the ones who had ED manipulation, 63 patients (86.31%) had an acceptable reduction and were discharged from the clinic without a follow-up and without any further intervention. A total of 10 patients (13.69%) underwent a repeat procedure in the theatre under general anaesthesia for unacceptable reduction within a two-week period.

In contrast, 72 children were included in the pre-COVID cohort (2019-20). The mean age at the time of injury was 9.2 years. The cohort had 41 males and 31 females. A total of 23 patients out of 72 patients (31.94%) had fracture manipulation in the ED. Among the ones who had ED manipulation, 12 patients were discharged from the clinic without a follow-up and 11 patients had a re-surgery under general anaesthesia within a two-week period. A total of 49 out of 72 patients (68.06%) had no attempt of closed reduction in the ED and were admitted for the general anaesthesia procedure in the theatre with no reasons documented and no formal Trust guideline in place. Of eligible patients in both cohorts, none had bilateral injuries. Comparisons between the two cohorts are summarized in Tables 1-2. Table 2 shows that the most common fracture subtype in both cohorts was metaphyseal fracture.

**Table 1:** Demographic data for the 2019-20 and 2021-22 cohorts

	2019-20 (pre-COVID)	2021-22
Age, years (mean)	9.2	9.8
Gender (male:female)	41:31 (n=72)	51:36 (n=87)

**Table 2:** Fracture types in the 2019-20 and 2021-22 cohorts

Fracture type	2019-20 (pre-COVID), n	2021-22, n
Salter-Harris fracture	23	27
Metaphyseal fracture (including off-ended)	28	35
Greenstick fracture	21	25
Total	72	87

Table 3 demonstrates a significant increase in ED fracture manipulation since the introduction of the new BOAST guidelines.

**Table 3:** Comparison of ED fracture manipulations between patients in the 2019-20 and 2021-22 cohorts (complex fractures excluded) *p-value was calculated using Fisher’s exact test [10].

	2019-20 (pre-COVID)	2021-22	p-value*
Fracture manipulated in the ED (yes:no)	23:49	73:14	<0.00001
Total, n	72	87	

Of the 87 patients in the 2021-22 cohort, 14 were not manipulated in the ED. The reasons for admission without a trial of manipulation was not documented in the notes. Amongst them, seven had off-ended distal radius fractures, three had displaced Salter-Harris fractures and four had angulated greenstick fractures. A total of 10 patients had failed ED manipulation in the 2021-22 cohort. These patients had admission for a theatre procedure. Among them, six had off-ended distal radius fractures, one had an unacceptable volar subluxed Salter-Harris fracture, two had unacceptable angulated greenstick fractures and one had an angulated metaphyseal fracture.

As demonstrated in Table 4, 63 out of the 87 patients had successful fracture reduction in the ED and admission was avoided. They were subsequently seen in the clinic and were discharged.

**Table 4:** Failed fracture manipulation in the ED for secondary procedures within two weeks (complex fracture pattern excluded) *p-value was calculated using Fisher’s exact test [10].

	2019-20 (pre-COVID)	2021-22	p-value*
Failed manipulation in the ED (yes:no)	11:12	10:63	0.0013
Total fractures manipulated in the ED, n	23	73	

Table 5 shows that although there was high compliance with limb neurovascular documentation and pre- and post-manipulation radiographs, less than 50% of patients had a follow-up within seven days demonstrating some room for improvement with respect to compliance with the BOAST guidelines.

**Table 5:** BOAST standards and compliance for the 2021-22 cohort BOAST, British Orthopaedic Association Standards for Trauma and Orthopaedics

Standard	Compliance (%)
Limb neurovascular status documentation pre-manipulation	93.10% (81/87)
Limb neurovascular status documentation before discharge	64.36% (56/87)
Consent documentation in the notes	73.56% (64/87)
Orthogonal X-rays pre- and post-manipulation	100% (87/87)
Fracture clinic appointment within 7 days	49.42% (43/87); average duration = 8.25 days

## Discussion

COVID-19 has undoubtedly caused a major shift in clinical practice across all specialities. It resulted in a push towards non-operative management in Trauma and Orthopaedics and a push towards telemedicine, to reduce the risk of transmission and reduce the operative and cost burden on the healthcare systems [11,12]. This shift in practice, due to pandemic and related restrictions, helped in re-discovering the forgotten art of non-operative treatment of fractures. Several studies have shown that early management of DRFFs in children can be safely done with optimum analgesia in the ED; it has been showed to reduce the anaesthetic and operative risk with equivalent reduction obtained using a general anaesthetic in the operation theatre. This treatment has been shown to be cost effective, reduce waiting times in the ED, and save space on already overstretched trauma operative lists, thus reducing strain on bed capacity. This is particularly important in National Health Services (NHS), United Kingdom.

However, there is a wide disparity in clinical practice among the various NHS Trusts, due to the lack of consensus guidelines at a national level [13,14]. In response to this, the British Orthopaedic Association published BOAST guidelines recommending early definitive management of these common injuries. At our Trust, prior to these guidelines, there was no clear consensus between the ED and Trauma and Orthopaedics department. The practice was mostly subjective with a high rate of admission and intervention in the operating theatre adding to the already overburdened trauma service. The development and implementation of the Trust guideline in accordance with the BOAST recommendations in our hospital helped in reducing waiting times for patients by avoiding unnecessary admissions and brought some uniformity among the practitioners and departments.

The main finding of our study was that through the implementation of a Trust pathway and adherence to national guidelines, the practice can be improved, resulting in reducing waiting times and avoiding unnecessary admissions for procedures in the theatre. Our findings also suggest that this can be safely done without any major complications.

The early fracture manipulation rate improved from 31.94% to 86.31%. The failed manipulation rates reduced from 47.82% (11 out of 23) to 13.69% (10 out of 73), when compared to the historical (pre-COVID) cohort. This was only possible with education and training. The success rates of fracture manipulation seemed to have improved due to more frequent procedures and involvement of teams.

The most common reason for not attempting reduction in the emergency department as well as for the failed manipulation is off-ended (complete translation) distal metaphyseal fractures. They are complex fractures requiring a complex reduction manoeuvre requiring sedation or a general anaesthetic for adequate pain control and muscle relaxation. In our study, all the six patients who underwent closed manipulation in the ED with diamorphine and Entonox for the off-ended distal radius fractures had unacceptable fracture reduction and underwent surgery in the operating theatre. Our findings are consistent with the results of a recent study that demonstrated that completely translated fractures are six times less likely to undergo a successful closed reduction [15]. Following this audit, the local protocol has been changed to manipulate these specific fractures under sedation.

This increase in achieving successful reduction in a clinical rather than operative setting has resulted in a significant reduction in seemingly unnecessary admissions and saved approximately 63 hours of theatre time (assuming 60 minutes per patient for anaesthesia and manipulation under anaesthesia). The mean theatre cost per hour is approximately £1200 [16]. The equivalent financial savings for 63 hours is approximately £75,600. This excludes any additional time, cost of implants and ward bed charges. Additionally, this theatre space helps in clearing trauma patients awaiting definitive surgery at home [17].

Although the BOAST guideline compliance was largely met in 2021-22, there were some categories that require improvement. We were 100% compliant with performing pre- and post-manipulation orthogonal radiographs and 93.10% compliant with documenting limb neurovascular status pre-manipulation; however, we were successful by 64.36% at documenting limb neurovascular status prior to discharge and had 73.56% compliance with documenting informed parental consent. Fortunately, any deviations from the standard protocol did not result in any actual patient harm.

The updated BOAST guidelines require follow-up within the seven days of discharge for all distal forearm fracture patients. Due to the high patient volume at our already strained trauma fracture clinic, we were only able to achieve 49.42% compliance. On analysis of patient outcomes, no circumstances were found that indicated worse patient outcomes or delayed surgery for the treatment of these complications.

There were also some limitations to this study. First, it suffers from the typical limitations of a retrospective cohort study, such as inability to determine confounding variables and missing data due to reliance on manually entered patient notes. Our minimum follow-up time was 11 months; however, there is potential for complications occurring past this time frame. Another limitation is the lack of patient-reported outcomes on pain relief, whole experience of ED manipulation, functional scores and parent satisfaction. However, a study from another UK hospital showed high parent satisfaction for distal forearm fractures managed in the ED [18].

## Conclusions

In this study, through data analysis of a retrospective cohort of paediatric distal radius fractures treated at a DGH in the UK, we have demonstrated that by implementing a local policy in accordance with the BOAST guidelines for the early management of paediatric forearm fractures, clinical practice can be improved by avoiding unnecessary admissions for surgery, saving trauma theatre time and cost for the trust. The audit data analysis has shown that although the busy DGH was unable to meet BOAST guidance for fracture follow-ups, this did not result in any adverse outcomes. Incidentally, we also found more uniformity in practice in our trauma service among the orthopaedic and emergency teams along with establishing a national consensus in practice.
